# Probing dopant segregation in distinct cation sites at perovskite oxide polycrystal interfaces

**DOI:** 10.1038/s41467-017-01134-x

**Published:** 2017-11-10

**Authors:** Hye-In Yoon, Dong-Kyu Lee, Hyung Bin Bae, Gi-Young Jo, Hee-Suk Chung, Jin-Gyu Kim, Suk-Joong L. Kang, Sung-Yoon Chung

**Affiliations:** 1 Department of Materials Science and Engineering, Korea Advanced Institute of Science and Technology (KAIST), 291 Daehak-ro, Yuseong-gu, Daejeon 34141 Korea; 2 Graduate School of EEWS, Korea Advanced Institute of Science and Technology (KAIST), 291 Daehak-ro, Yuseong-gu, Daejeon 34141 Korea; 3 KAIST Analysis Center, Korea Advanced Institute of Science and Technology (KAIST), 291 Daehak-ro, Yuseong-gu, Daejeon 34141 Korea; 4 Jeonju Center, Korea Basic Science Institute, 20 Geonji-ro, Deokjin-gu, Jeonju 54907 Korea; 5 Korea Basic Science Institute, 169-148 Gwahak-ro, Yuseong-gu, Daejeon 34133 Korea; 6 Korea Institute of Ceramic Engineering and Technology, 101 Soho-ro, Jinju 52851, Korea

## Abstract

Although theoretical studies and experimental investigations have demonstrated the presence of space-charge-induced dopant segregation, most work has been confined largely to the crystal-free surface and some special grain boundaries, and to the best of our knowledge there has been no systematic comparison to understand how the segregation varies at different types of interfaces in polycrystals. Here, through atomic-column resolved scanning transmission electron microscopy in real polycrystalline samples, we directly elucidate the space-charge segregation features at five distinct types of interfaces in an *AB*O_3_ perovskite oxide doped with *A*- and *B*-site donors. A series of observations reveals that both the interfacial atomic structure and the subsequent segregation behaviour are invariant regardless of the interface type. The findings in this study thus suggest that the electrostatic potential variation by the interface excess charge and compensating space charge provides a crucial contribution to determining not only the distribution of dopants but also the interfacial structure in oxides.

## Abstract (web summary)

Space-charges in polycrystalline materials can drive segregation of dopants, however an in-depth understanding of this process is still missing. Here, the authors show that in polycrystalline perovskites the space-charge segregation and interfacial structure are nearly identical irrespective of the interface type.

## Introduction

Based on the different formation free energies between plausible point defects, the presence of surface excess charge and compensating subsurface space charge with an opposite sign in ionic crystals was initially suggested by Frenkel^1^ in 1946 and also by Lehovec^2^ in 1953, explaining the local nonstoichiometric distribution of point defects in the surface region. A series of follow-up studies by Kliewer and colleagues^3–6^ in the mid-1960s theoretically consolidated the existence of space charge and systematically calculated the variation of space-charge potential and the resulting distribution of vacancies and aliovalent impurities in ionic halide crystals by taking the long-range columbic interaction and even the electron affinity into account. Spurred by these milestone works, many notable experimental observations as well as theoretical calculations have been carried out in order to shed light on the dopant segregation behaviour and subsequent correlation with the space charge in ionic crystals^7–16^.

**Fig. 1:**
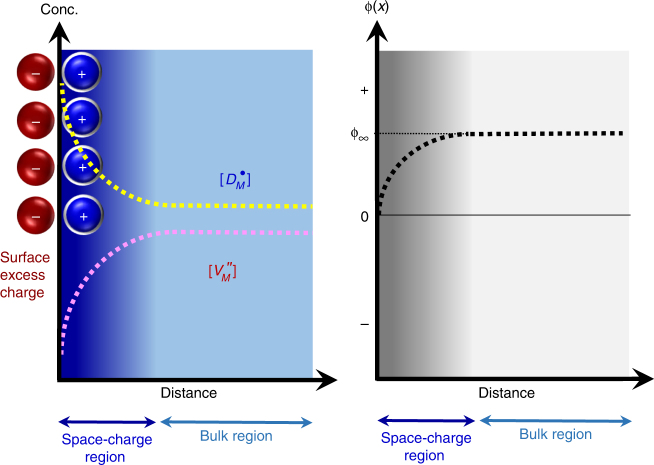
Distribution of charged defects and electrical potential near the surface. This figure shows the case of donor doping in a *M*O-type oxide crystal. $\left[D_{M}^{\cdot}\right]$ and $\left[ {V_M^\prime} \right]$ in the left diagram indicate the concentrations of donors and cation vacancies in the Kröger–Vink notation, respectively. The ϕ_∞_ in the right diagram also denotes the electrical potential in the bulk far from the surface. Strong segregation of donors and corresponding deficiency of cation vacancies in the space-charge layer is noted to compensate the excess surface charge. For more details, see Supplementary Note 1 and Supplementary Fig. 1

Figure 1 schematically depicts the concentration of charged defects, including donor dopants, and the electrical potential variation near the surface in oxides for donor doping as an example on the basis of the previous theoretical studies in general (see Supplementary Note 1 and Supplementary Fig. 1 for details)^3–7^. As described in this set of diagrams, the distribution of defects and the potential dramatically vary within a considerably thin space-charge layer, showing comprehensively different aspects compared with those in the bulk. Despite many experimental works on the space-charge-driven interface segregation via nanoscale point analyses and line scan^8–10^, most of them failed to clarify the variation of the chemical composition and the physical structure at the interface in detail. Without atomically resolved direct observation, it is very challenging to scrutinize the two distinct layers for the negative surface excess charge and the compensating positive space charge, as shown in Fig. 1.

Two significant issues have been overlooked for a long time regarding the space-charge-driven dopant segregation. One is how the segregation behaviour would vary between dopants occupied in two distinct sites in complex oxides, such as *AB*O_3_-type perovskites. For example, while both La^3+^ substituted for *A* ^2+^ and Ta^5+^ for *B* ^4+^ in *AB*O_3_ oxides identically act as a donor from a defect chemical viewpoint, their occupation is clearly distinguished in the dodecahedral *A* site and the octahedral *B* site, respectively. Nevertheless, little is known about how they are distributed in the space-charge region based on the different site occupancy. The other issue is the interface-type dependency of the segregation. It is noted that the initial theories proposed by Frenkel^1^, Lehovec^2^, and Kliewer and colleagues^3–6^ dealt with the free surface of crystals for simplicity. Even though the basic ideas derived for the free surface have been adopted to explain the overall segregation characteristics at other types of interfaces including grain boundaries, to the best of our knowledge no specific comparison of segregation between different interfaces has been made, and as a result there is a lack of understanding regarding the influence of the interface type and structure.

Here, by using polycrystalline perovskite (Ca_1/4_Cu_3/4_)TiO_3_^17–19^ as a model system in this study (see its crystal structure in Supplementary Fig. 2), we systematically provide a series of direct observations on notable space-charge segregation aspects at atomic resolution. As already demonstrated in other alloys and oxides^20–25^, the large size mismatch of dopants inducing substantial misfit strain energy is another major driving force for interface segregation^26, 27^. To focus on the space-charge effect and suppress the misfit-strain-induced segregation, we thus select Ta^5+^ (ionic radius, *r* = 0.64 Å) at Ti^4+^ (*r* = 0.61 Å) sites and La^3+^ (*r* = 1.36 Å) at Ca^2+^ (*r* = 1.34 Å) sites for substitution as two independent donor dopants, the size mismatch of which is <5%^28^. For direct atomic-column probing and chemical verification at the interface, scanning transmission electron microscopy (STEM) in both high-angle annular dark-field (HAADF) and bright-field (BF) modes is utilized along with energy-dispersive X-ray spectroscopy (EDS) based on the four integrated X-ray detectors for efficient collection of signals^29, 30^. Density-functional-theory (DFT) calculations are also performed by using the CASTEP code to verify which layer is energetically favourable for segregation at the {100} surface. The findings in our study demonstrate that the space-charge segregation behaviour at five distinct types of interfaces parallel to the {100} plane is nearly identical regardless of the interface type and thus highlight the impact of electrostatic potential on the structural and compositional variation in the interface region in perovskite oxides.

## Results

### Five types of interfaces in polycrystals

**Fig. 2:**
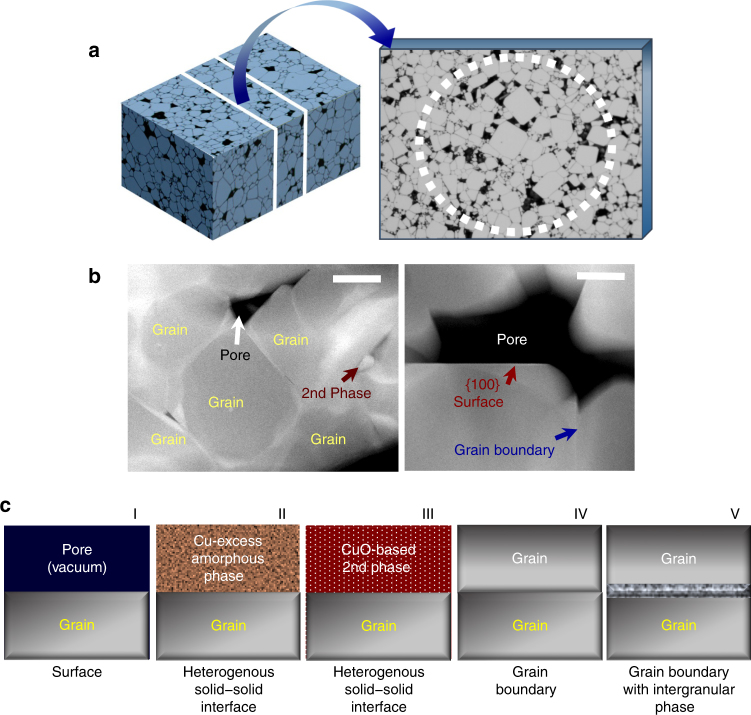
Polycrystalline microstructure and five distinct types of interfaces. **a** A typical ceramic sample consisting of numerous grains is schematically illustrated on the left-hand side of the figure. The sectioned thin specimen for STEM observation is also delineated as a magnified illustration. **b** HAADF-STEM images show the actual microstructure of our polycrystalline samples. The black image feature denotes pores in these dark-field images. Second phases with distinguishably brighter contrast are also observable in the left image. As shown in the right image, both a free surface in contact with a pore (red arrow) and solid–solid interfaces (blue arrow) are identified. Scale bars, 500 nm (left) and 200 nm (right). **c** Five possible types of interfaces in our polycrystalline sample are summarized in this set of illustrations on the basis of the observed microstructure, enabling a systematic structural comparison between the different types

As schematically illustrated in Fig. 2a, a sintered polycrystalline sample with a number of grains and pores contains various interface configurations, encompassing the free surface between a grain and a pore, a heterogeneous solid–solid interface between a grain and a second phase, and grain boundaries between individual grains^31^. It is considerably time consuming during STEM observation to identify suitable interfaces parallel to a low index plane in the polycrystalline microstructure consisting of numerous grains with a completely random orientation (see the crystallographic orientation map of grains and the orientation-distribution pole figure obtained in our polycrystalline (Ca_1/4_Cu_3/4_)TiO_3_ samples via the electron backscattered diffraction (EBSD) analysis in Supplementary Fig. 3). Despite this complexity, however, polycrystalline samples have a notably significant advantage over other types of specimens in that different interface configurations can be probed in a single sample at one time.

The HAADF-STEM images presented in Fig. 2b show typical microstructure observed in our samples. In addition to the presence of pores, Cu-excess secondary phases, which are either crystalline or amorphous, could be identified during the STEM analysis, as denoted by a dark red arrow in the left image in Fig. 2b (see a set of EDS maps provided in Supplementary Fig. 4 for the Cu-excess and Ca/Ti-deficient composition of the second phases). The existence of these Cu-excess secondary phases in spite of the initially stoichiometric composition of the samples, (Ca_1/4_Cu_3/4_)Ti_1−*x*_Ta_*x*_O_3_ and (Ca_1/4−*y*_La_*y*_Cu_3/4_)TiO_3_, strongly implies that the effectively positive charge of the donors, Ta and La, is largely compensated via the formation of the negatively charged Cu vacancies rather than free electrons, in good agreement with a previous impedance study^32^. Consequently, considering the presence of pores and second phases, five distinct interface configurations can be categorized for STEM observation, as clarified in Fig. 2c: grain surface in contact with a pore (Type I); heterogeneous solid interface between a grain and a Cu-excess amorphous phase (Type II); another heterogeneous solid interface between a grain and a Cu-excess crystalline phase (Type III); grain boundaries (Type IV); and grain boundaries with an intergranular amorphous layer (Type V). In particular, most of our observations deal with interfaces parallel to a {100} plane for direct comparison of atomic structure and compositions between different types of interfaces.

### Observation of surface (Type I)

**Fig. 3:**
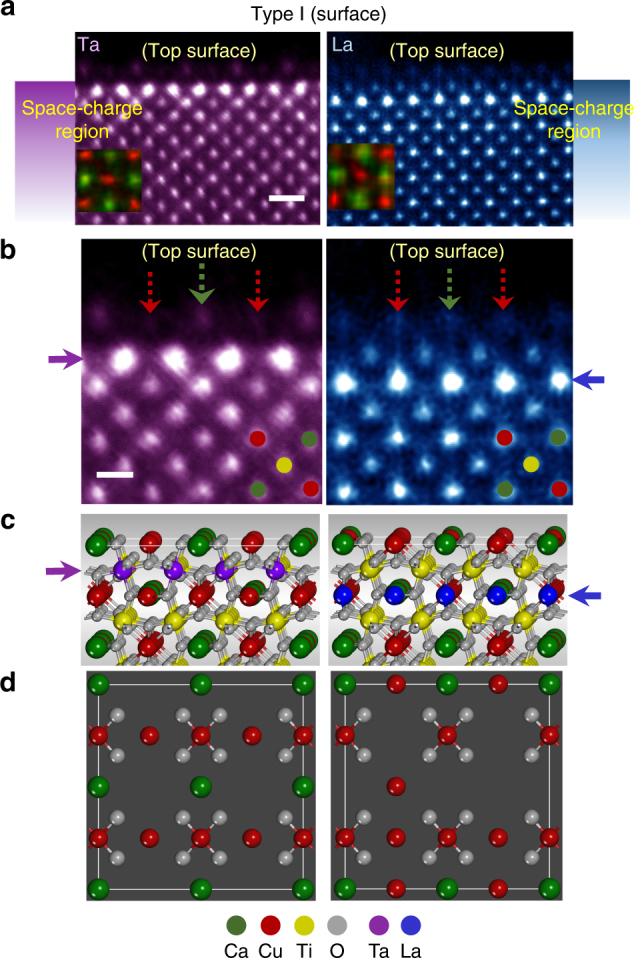
Structures of Type-I {100} interfaces (free surface). **a** HAADF images from Ta- and La-doped samples are provided. The insets superimposed on the images are atomic-scale composition maps discriminating the ordered array between Ca (green) and Cu (red) in the *A* sites. Scale bar, 5 Å. **b** The enlargements are given to compare the different positions of a segregation layer, as denoted by purple and blue arrows, respectively. However, the top surface is noted to identically consist of Ca- and Cu-column termination in both cases, as indicated by green and red arrows. Scale bar, 2.5 Å. **c**, **d** Each set of illustrations describes the plausible atomic structure in the cross-sectional view in (**c**) and in the (100) plane view in (**d**)

Figure 3a presents a pair of HAADF-STEM images exhibiting typical atomic structures at the {100} free surface (Type I) of grains. The inset in each of the images is an atomic-column resolved composition map of Ca (red) and Cu (green) obtained from the EDS analysis. Therefore, the ordered array of Ca and Cu in the *A* site is easily discriminated in addition to the *B*-site Ti columns (see the crystal structure in Supplementary Fig. 2). Owing to the much higher atomic number (Z) of Ta and La than that of the matrix cations, bright Z-contrast image features are readily observable beneath the surface in both Ta- and La-doped samples, directly demonstrating the strong dopant segregation. As revealed more clearly in the enlargements in Fig. 3b, the top-most surface layer and the subsurface segregation layer with distinct bright contrast in the space-charge region are distinguished from each other in good agreement with a previous report^13^.

Layer-by-layer scrutiny of this set of atomic-scale images in Fig. 3b allows us to unveil several notable aspects in the segregation. First, distinct subsurface layers of segregation in the space-charge region are clearly identified, depending on the substitutional site by dopants. Ta in the Ti site strongly segregates in the second layer from the top surface (purple arrow in the left image). In contrast, La in the Ca site segregates in the third layer (blue arrow in the right image). Atomistic illustrations in Fig. 3c schematically depict the different position of Ta- and La-segregation layers for better visualization. This comparison via atomic-column images offers the first direct evidence proving that the location of space-charge dopant segregation can vary in multicomponent complex oxides even if the effective charges of dopants are identical to each other. Second, while most Ta dopants maintain the Ti-site occupation in the subsurface segregation layer (purple arrow), La appears to be substituted for both Ca and Cu in the segregation layer (blue arrow), showing no preferential occupancy in the Ca site, in contrast to the observation in the bulk^33^.

Another noticeable feature observed in Fig. 3a, b is that the *A*-site termination of the {100} top surface comprising Ca and Cu columns does not change in either of the samples, regardless of the added dopants. Red and green arrows on the top surface in both images in Fig. 3b indicate Cu and Ca/Cu columns, respectively. As denoted by red arrows for the Ta-doped sample, the Cu columns in the top-surface layer show a significantly low intensity, directly demonstrating the presence of Cu vacancies. Therefore, the cation-deficient surface with an effectively negative charge agrees well with the positively charged donor segregation as a compensating space charge in the subsurface layers in accord with a previous report^13^. In contrast to the Ta-doped grains, such a periodic intensity variation between Cu columns at the top surface of grains is not observed in the La-doped sample, as can be found in the right panel in Fig. 3a, b. As a result, a random distribution of cation vacancies at the surface is anticipated. To delineate a plausible atomic structure with a different cation-vacancy distribution at the surface for each case, planar-view schematic illustrations are provided in Fig. 3d. An atomic-scale EDS analysis also has been carried out for chemical verification regarding the *A*-site termination at the surface as well as the subsurface segregation of Ta and La. See Supplementary Figs. 5 and 6 for details on the analysis results.

### Observation of heterointerfaces (Types II and III)

**Fig. 4:**
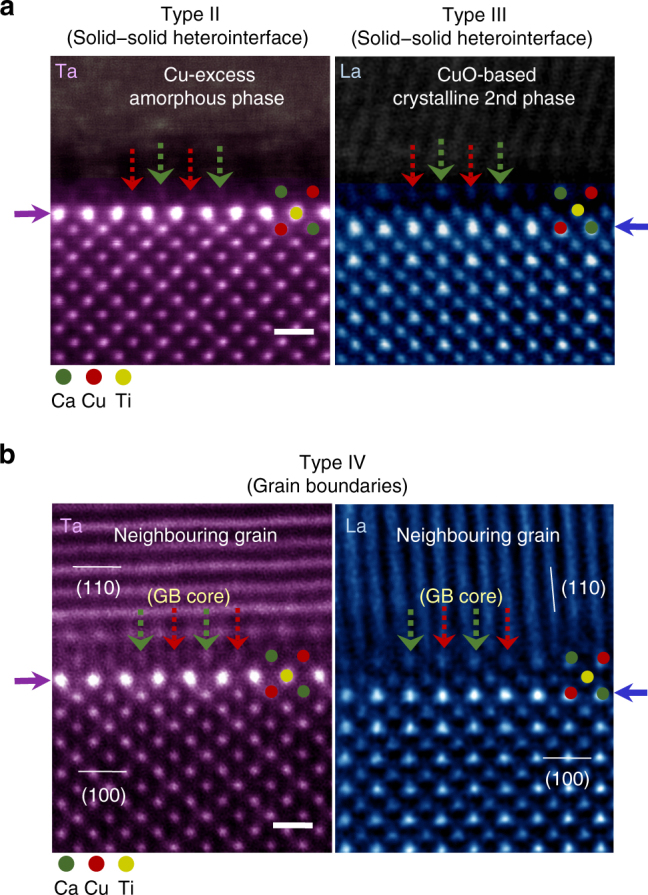
Structures of Type-II, Type-III, and Type-IV {100} interfaces. **a** Two different solid–solid heterogeneous interfaces (Types II and III) are demonstrated, revealing nearly identical atomic configurations at the interface cores (green and red arrows) and segregation aspects (purple and blue arrows) to those observed at the surface. Scale bar, 5 Å. **b** The grain-boundary cores in both samples also consist of the *A*-site-cation Ca and Cu columns. In addition, no variation is observed in the position of a strong segregation layer. Therefore, this series of HAADF images supports the notion that the atomic structure and space-charge segregation are not seriously influenced by the interface type. Scale bar, 5 Å

The most important and unexpected finding during the STEM observation in our study is that the aforementioned space-charge segregation behaviour and the atomic structure shown at the top surface are not affected by the type of interface and rather notable similarities are revealed among the five distinct types in each of the samples. Although the composition of the second phases is analogously Cu excess in both samples, as already demonstrated in the EDS maps in Supplementary Fig. 4, an amorphous form is found in the Ta-doped sample, in contrast to a crystalline phase in the La-doped sample. Consequently, typical atomic structures of both Type I and Type III interfaces could be observed in each of the samples. As denoted by red and green arrows in HAADF-STEM images in Fig. 4a, the interface core between a {100} grain and a second-phase particle consists of Ca- and Ca/Cu columns in both cases, exhibiting an identical *A*-site termination structure with that of the top-most surface shown in Fig. 3. It is also noted that the subsurface segregation layer of Ta and La places in the same position, the second layer (Ti columns) for Ta and the third layer (Ca and Cu columns) for La from the interface core, as indicated by purple (left image) and blue (right image) arrows in Fig. 4a.

### Observation of grain boundaries (Types IV and V)

Such locations of space-charge segregation and atomic structure at the interface core discovered in Types I–III were identified to be invariant in general even at grain boundaries (Type IV). Figure 4b shows the grain boundaries, at which the [001] zone of one of the two adjacent grains is parallel to the electron-beam direction in STEM so as to make a direct comparison of the atomic-scale structure with that of other types of interface. As clearly verified in this set of images, each of the grain-boundary core is composed of Ca and Ca/Cu columns in the *A* site, accompanying the dopant segregation to construct the space charge nearby. A series of EDS-based chemical analysis results for Types II–IV is provided in Supplementary Figs. 7−9 to confirm the dopant segregation in the space-charge layers.

Amorphous phases in the triple pockets in polycrystalline ceramics can penetrate along grain boundaries when relevant thermodynamic conditions, including the wettability, are satisfied^34–40^. Since the theoretical milestone work by Clarke^34^ in 1987, the presence of nm-thick intergranular films at grain boundaries has been proved to be one of the thermodynamically stable configurations in numerous polycrystalline ceramic materials encompassing nitrides, carbides, oxides, and even complex perovskites^35–40^. Therefore, as a Type-V interface, the grain boundaries wet by nanoscale intergranular amorphous films can form in the Ta-doped sample where a Cu-excess amorphous phase is frequently observed in the triple pockets.

**Fig. 5:**
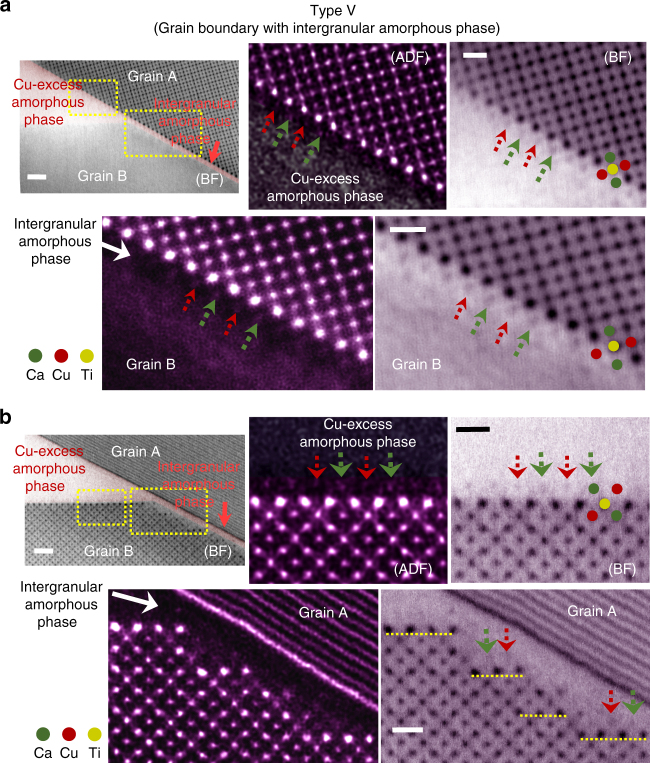
Structure of Type-V interface. An amorphous phase in the triple pocket wets a grain boundary, forming a nm-thick intergranular amorphous layer. Therefore, both a Type-II solid interface and a Type-V grain boundary can be observed at the same time. **a** These sets of images in HAADF and bright-field modes provide enlargements for the two locations indicated by yellow rectangles, when the (001) zone of Grain A is aligned to the electron beam direction. It is noted that the identical atomic structure and segregation are commonly found even in Type-V interface. Scale bars, 1 nm (left) and 5 Å (right). **b** These sets of images also show the same location. As the (001) zone of Grain B is aligned, the atomic-column resolved image feature can be acquired on the Grain-B side. In addition to the consistent results shown at the Type-II interface (upper row), the yellow dashed lines in the bright-field image in the lower row denote the stepwise Ta segregation along the small facets. Scale bars, 1 nm (left) and 5 Å (right)

Low-magnification BF images in Fig. 5a, b indeed show the typical morphology regarding the penetration of a thin amorphous phase from the triple pocket along a grain boundary. As the images in Fig. 5a were acquired after the [001] zone of Grain A was aligned to be parallel to the electron beam direction, atomic columns in Grain B were not imaged. Two pairs of HAADF and corresponding BF images shown in Fig. 5a are enlargements of the regions denoted by yellow rectangles, simultaneously demonstrating both the Type-II interface and the Type-V grain boundary, respectively. In agreement with the observation at the Type-II interface (upper images), the discrimination of the *A*-site cation termination of Grain A (red and green arrows) even in this very thin amorphous layer is one of the notable findings in the atomic structure at the Type-V grain boundary (lower images). Similarly, Fig. 5b shows pairs of magnified STEM images for the two regions denoted by yellow rectangles when the [001] zone of Grain B is aligned to the electron beam direction so as to enable atomic-column visualization for the side of Grain B at this Type-V grain boundary. As easily recognized in the lower images, nanoscale {100} facets on Grain B are developed along the grain boundary. Although these facets are of a very small length scale, the consistent *A*-site cation termination (red and green arrows) and the stepwise underneath segregation of Ta (yellow dashed lines) parallel to each of the facets, as indicated in the BF image more clearly, are other noticeable aspects in this set of STEM images in Fig. 5b.

**Fig. 6:**
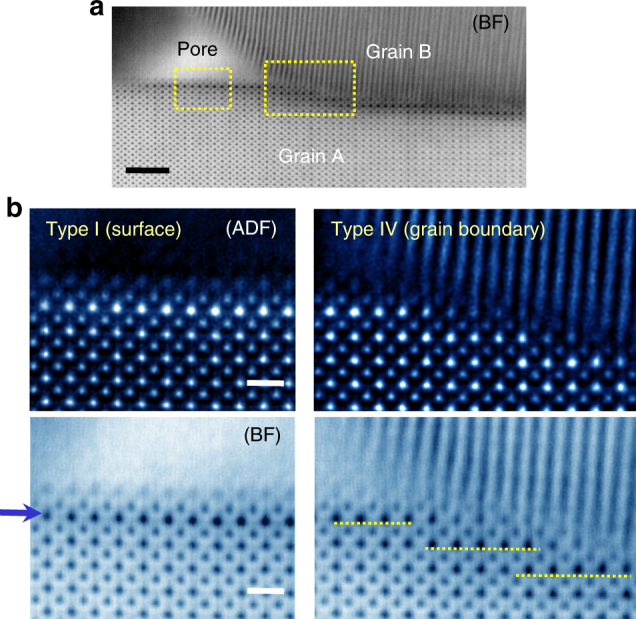
Interfaces in a triple-junction region in the La-doped sample. **a** This bright-field image presents the typical morphology of a nanoscale pore and a contacting grain boundary. As indicated by yellow rectangles, both the Type-I surface and the Type-IV grain boundary are simultaneously captured in the image. Scale bar, 2 nm. **b** Two sets of magnified HAADF and bright-field images are provided for the denoted locations in (**a**). In addition to consistent structural features with the observations in Fig. 3 being verified at the Type-I surface (left-hand panel), analogous stepwise La segregation along the {100} facets (yellow dashed lines) is also noted at the Type-IV grain boundary (right-hand panel). Scale bars, 5 Å

The BF image in a low magnification in Fig. 6a shows the overall morphology of a pore in the triple pocket along with a grain boundary between Grains A and B, enabling observation of a Type-I surface and a Type-IV grain boundary at the same time. As magnified in the left-hand side in Fig. 6b, the surface structure and the subsurface La segregation (blue arrow in the BF image) can be verified to be identical to those shown in Fig. 3. In addition, the yellow dashed lines in the BF image in the right-hand side consistently indicate the same stepwise segregation behaviour in the {100} vicinal plane along the Type-IV grain boundary, consistent with the observation in Fig. 5b.

## Discussion

**Fig. 7:**
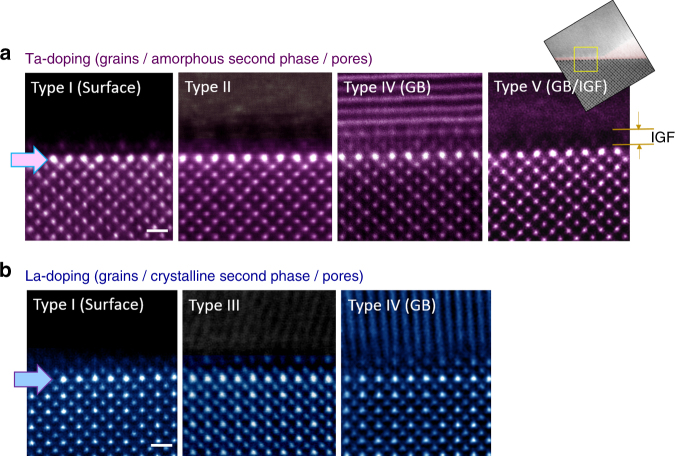
Direct comparison between five different types of interfaces. **a** Strong Ta segregation is clarified in the vicinity of the interface core in each case as a common feature for the Ta-doped sample (upper row). A nm-thick intergranular film (IGF) is denoted by a pair of orange arrows on the image of the Type-V grain boundary. Scale bar, 5 Å. **b** La enrichment at the *A*-site layer underneath each of the interfaces is also noted for the La-doped sample (lower row), verifying the space-charge-driven segregation behaviour. Scale bar, 5 Å

Figure 7 summarizes all the STEM observations carried out in this work for direct comparison of the {100}-plane interface structure and the segregation behaviour between the different types of interfaces. Note that Type III does not exist in the Ta-doped sample because the Cu-excess second phase is amorphous and, similarly, neither Type II nor IV is present in the La-doped sample as the secondary phase is crystalline. As systematically demonstrated in this set of images, both the structure of the top surface and the interface core and the underneath segregation behaviour in the space-charge layer are nearly identical in all types of interfaces in each of the doping cases, showing significant insensitivity to the type of interfaces. Extra sets of STEM images showing the same results are provided in Supplementary Fig. 10 to clarify the generality of our observations in the polycrystalline samples. Our DFT calculations also consistently verify that both Ta and La have a strong tendency for subsurface segregation at the {100} surface (see Supplementary Fig. 11). In particular, the calculation result revealing that the lowest energy value is obtained when La segregates in the subsurface layer, not on the top-most surface, critically supports our experimental observations (see Fig. 7b). This confirms that the variation in electrostatic potential near the interface has a profound impact even on the segregation behaviour in the space-charge region.

Focusing on the {100}-orientation interfaces by atomic-scale scrutiny in polycrystalline (Ca_1/4_Cu_3/4_)TiO_3_ ceramic samples doped with Ta and La, we have successfully elucidated the critical details regarding the atomistic structure and dopant segregation at five different types of interface with a {100} orientation. Regardless of the interface type, the outmost layer of the {100} plane of grains was found to always consist of negatively charged Ca and Cu termination. In addition, to compensate the negative charge from the interface core (or the top surface), La was observed to segregate among the *A*-site cations in the space-charge region. Visualizing atomic column-by-column structures at possible configurations of crystal interface, the direct observations in this work also emphasize the crucial influence of electrostatic potential on both the physical structure and the chemical composition including segregation in the interface region in perovskite oxides.

## Methods

### Powder synthesis and fabrication of polycrystals

Before fabricating polycrystalline dense samples, Ta- and La-doped (Ca_1/4_Cu_3/4_)TiO_3_ (equivalently, CaCu_3_Ti_4_O_12_) powders were first synthesized via a conventional mixed oxide technique using CaCO_3_ (99.995%, Aldrich), CuO (99.995%, Alfa Aesar), TiO_2_ (99.8%, Aldrich), Ta_2_O_5_ (99.9%, Aldrich), and La_2_O_3_ (99.99%, Aldrich). The initial stoichiometry of 5%-Ta-doped powder was adjusted to be (Ca_1/4_Cu_3/4_)Ti_0.95_Ta_0.05_O_3_ for the *B*-site doping. Based on a previous report showing the La substitution in the Ca site^33^, a La-doped stoichiometric powder of (Ca_0.20_La_0.05_Cu_0.75_)TiO_3_ was prepared for 5% *A*-site doping. Both powder mixtures of the starting materials were ball-milled in ethyl alcohol for 24 h. The dried slurries were then calcined at 900 °C in air for 12 h. The calcined powders were ball-milled again for another 2 h to obtain fine particles. The Ta- and La-doped powders were both slightly pressed into disks and then isostatically pressed under 200 MPa to fabricate polycrystalline samples. The pellets were sintered in a tube-type furnace at 1150 °C in air for 12 h, and removed immediately to room temperature without any furnace cooling to obtain high-temperate equilibrium segregation.

### STEM and EDS

Samples for STEM observation were prepared with densely sintered polycrystals by mechanical grinding to a thickness of 80 μm, dimpling to a thickness of <10 μm, and ion-beam thinning for electron transparency. Bright-field STEM and Z-contrast HAADF-STEM images were taken with a transmission electron microscope (Titan cubed G2 60-300, FEI) at 300 kV with a spherical aberration corrector (CEOS GmbH). The optimum size of the electron probe was ~1 Å with a convergence semiangle of 24 mrad. The collection semiangles of the HAADF detector were set to be 40−200 mrad in order to exploit large-angle elastic scattering of electrons for clear Z-sensitive images. The obtained raw images were band-pass filtered to reduce background noise. Atomic-scale chemical-composition mapping by EDS was performed in the same transmission electron microscope operated at 300 kV. As four integrated silicon-drift EDS detectors (ChemiSTEM™ Technology) were positioned around a specimen with a collection solid angle of 0.7 sr, much higher count rates of X-ray signals in shorter time could be achieved. Ca-K_α_ (3.7 keV), Cu-K_α_ (8.0 keV), Cu-L_α_ (0.9 keV), Ti-K_α_ (4.5 keV), and Ta-M_α_ (1.7 keV) lines were selected during atomic-column mapping. Because the La-M_α_ (0.84 keV) line seriously overlaps with the Cu-L_α_ (0.9 keV), the La-L_α_ (4.65 keV) line was utilized instead during the analysis of a La-doped sample. As shown in Supplementary Fig. 6, our EDS detector has a sufficient energy resolution (~0.1 keV) in a signal spectrum and thus clearly distinguishes the La-L_α_ line from the Ti-K_α_ line, enabling the chemical verification of La in the sample. To reduce electron-beam broadening effects, the probe current was adjusted to be 30−50 pA with a scanning time of <200 s. The EDS maps were low-pass filtered using Bruker Esprit software after reduction of background noise for better visualization.

### SEM and EBSD

The polycrystalline microstructure after fine polishing was examined in a scanning electron microscope (Quanta 3D FEG, FEI) at 30 kV. To determine the crystallographic orientation of each grain, an EBSD analysis was carried out by using a high-resolution EBSD camera (Hiraki Pro, EDAX) with the OIM™ data collection and analysis software packages (EDAX/TSL). Based on the orientation information between grains, the grain-boundary misorientation angle could be estimated. More than 90% of grain boundaries examined during the EBSD analysis were identified to be high-angle random boundaries.

### DFT calculations

Ab initio DFT calculations were carried out within the spin-polarized generalized-gradient approximation along with the PBEsol functional revised for exchange correlation of densely packed solids and the ultrasoft pseudopotentials for ionic cores, as implemented in the CASTEP code (Biovia). A sufficiently long slab was constructed along (more than 8 unit cells in length) with a 10 Å vacuum layer as an optimum supercell for each calculation to make the relaxation layer of each slab >20 Å in thickness, as previously reported^13^. Based on the STEM observations, the [CaCu_2_O_4_] unit was adopted for the top surface structure of the supercell, as shown in Fig. 2d. To compare the relative lattice stability depending on the dopant segregation positions in the surface region, the energy difference, Δ*E*_seg_, is defined as follows.

Δ*E*_seg_ = *E* (dopant in the *n*th Ca or Ti layer from the surface) – *E* (dopant in the fourth Ca or Ti layer).

Where *E* is the lattice enthalpy of a supercell containing dopants. When dopants are in the fourth Ca or Ti layer, Δ*E*_seg_ is zero as a reference. Consequently, a more negative value of Δ*E*_seg_ represents an energetically more favourable configuration of dopants. The plane-wave basis set for a kinetic energy cutoff was 440 eV. Full relaxation of the internal coordinates for each atom in the relaxation layer was performed using the BFGS (Broyden–Fletcher–Goldfarb–Shanno) algorithm with convergence tolerances of 0.1 eV Å^−1^ for the maximum ionic force, 5 × 10^−5^ eV per atom for the total energy, and 0.005 Å for the maximum ionic displacement.

### Data availability

The data that support the findings of this study are available from the corresponding author (S.-Y.C.) on reasonable request.

## Electronic supplementary material

Supplementary Information
