# Supplementary material for: Probing dopant segregation in distinct cation sites at perovskite oxide polycrystal interfaces
Source: Nat Commun. 2017 Nov 10;8:1417. doi: 10.1038/s41467-017-01134-x (PMC5681544; doi:10.1038/s41467-017-01134-x)
Supplement: Supplementary file 1 — Supplementary Information [file 41467_2017_1134_MOESM1_ESM.pdf]

Supplementary Figures

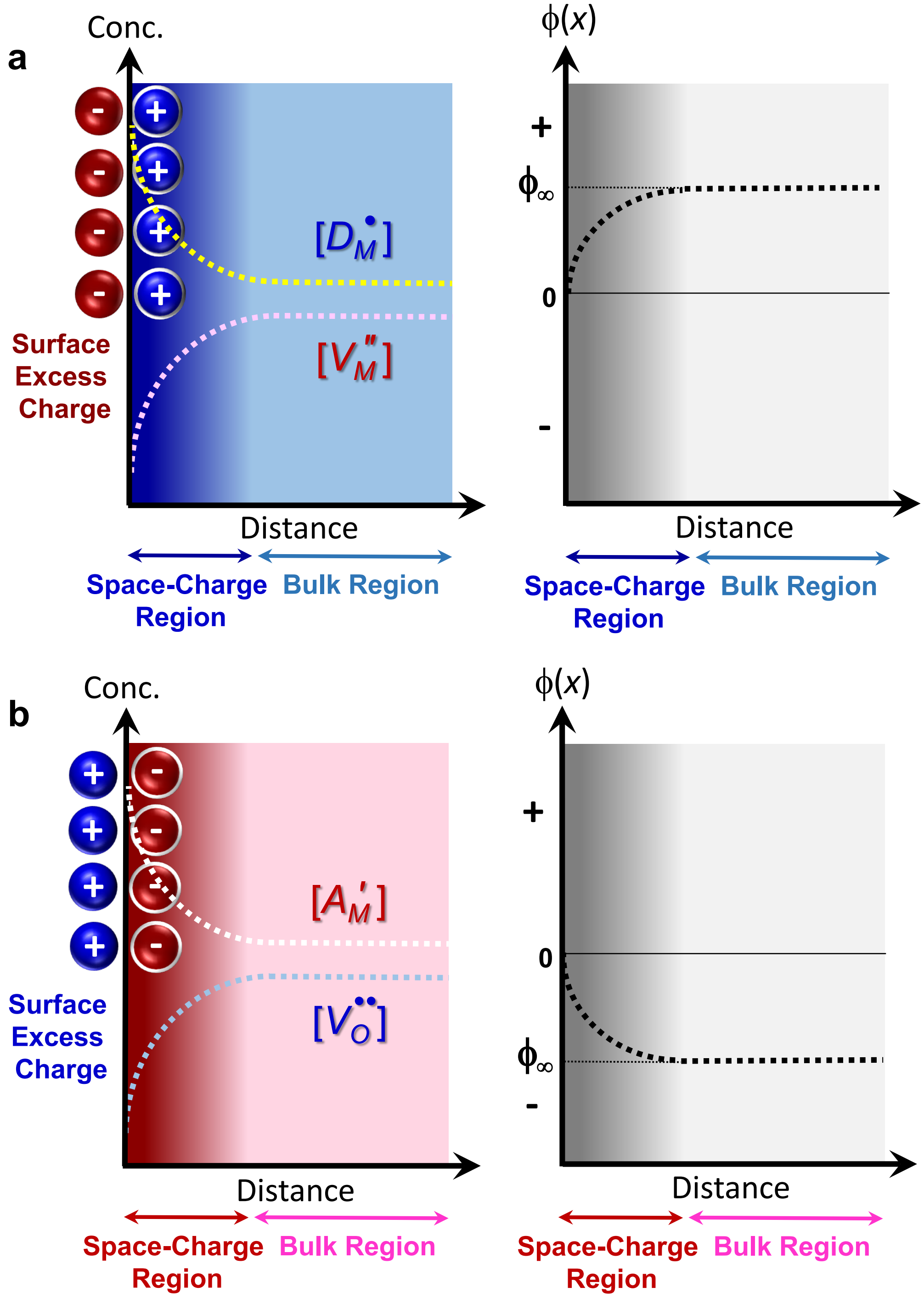

Supplementary Fig. 1 Distributions of charged defects in a *M*O-type oxide. Dopants (left) and relevant electrical potential variations (right) near the surface are presented. Schematic diagrams for (**a**) donor-doped and (**b**) acceptor-doped cases are illustrated, respectively. It is noted that the exponentially varying concentrations of dopants and oppositely charged vacancies in the space-charge are established to compensate the excess charge at surface. The general concepts of the space-charge theory and other details are described in Supplementary Note 1.

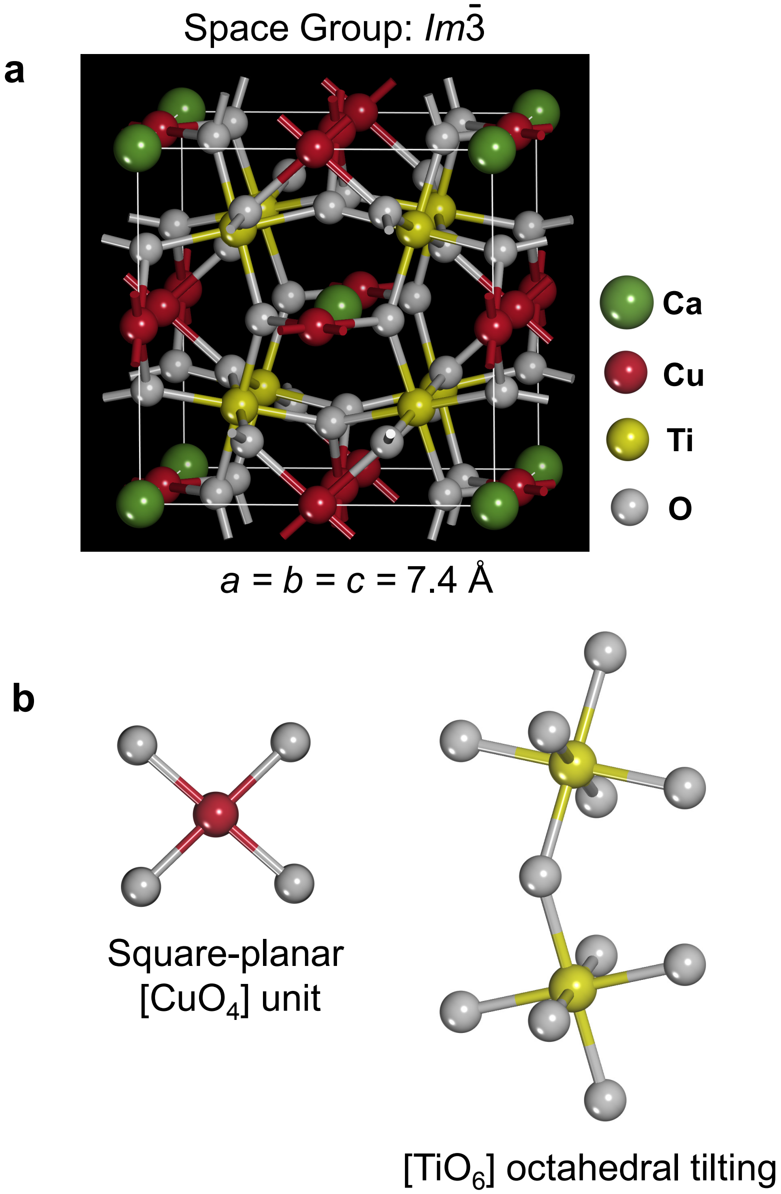

Supplementary Fig. 2 Crystal structure of $(Ca_{1/4}Cu_{3/4})TiO_3$ (equivalently, $CaCu_3Ti_4O_{12}$). **a** The ordered array between Ca and Cu in the *A* site of the *AB*O_3-type perovskite framework is revealed to be a noticeable structural aspect in this crystal. **b** The occupation by relatively small Cu^{2+} cations in the *A* site thus results in the octahedral tilting (Glazer's tilt symbol: $a^+a^+a^+$) of [TiO_6] as well as the square-planar coordination of Cu with oxygens in a body-centered cubic structure.

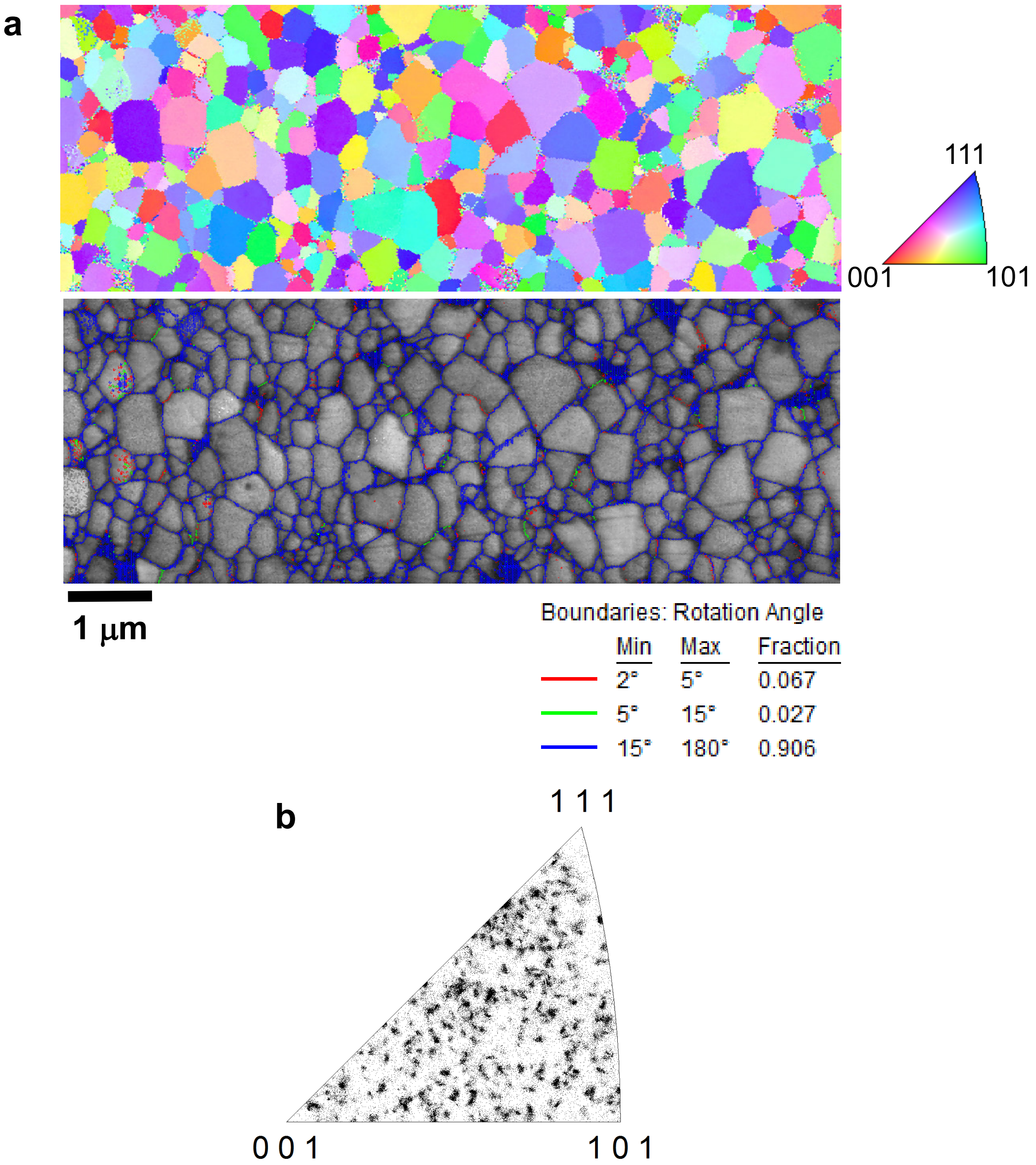

Supplementary Fig. 3 Analysis results from the electron backscatter diffraction (EBSD). **a** The crystallographic orientation map for grains is shown in color in the upper row, demonstrating that each grain has no preferential orientation. The grain-boundary misorientation angles obtained from the EBSD are also denoted by three different colors (red, green, and blue) on the SEM image in the lower row. As clarified in the summary, more than 90% of the grain boundaries are high-angle (>15°) boundaries (blue lines). **b** The random orientation of grains can be confirmed by this pole figure, which reveals no preferred orientation distribution.

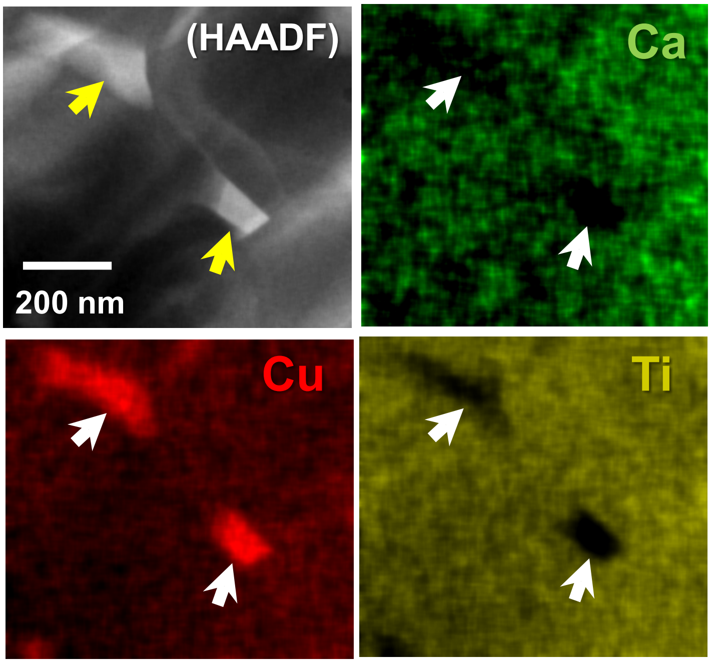

Supplementary Fig. 4 EDS chemical composition maps showing the Cu-excess second phases. As indicated by yellow arrows in the HAADF image, secondary phases having a brighter contrast are frequently observed in both Ta- and La-doped samples during the STEM analysis. The white arrows in each of the EDS maps verify the enrichment of Cu together with the deficiency of Ca and Ti.

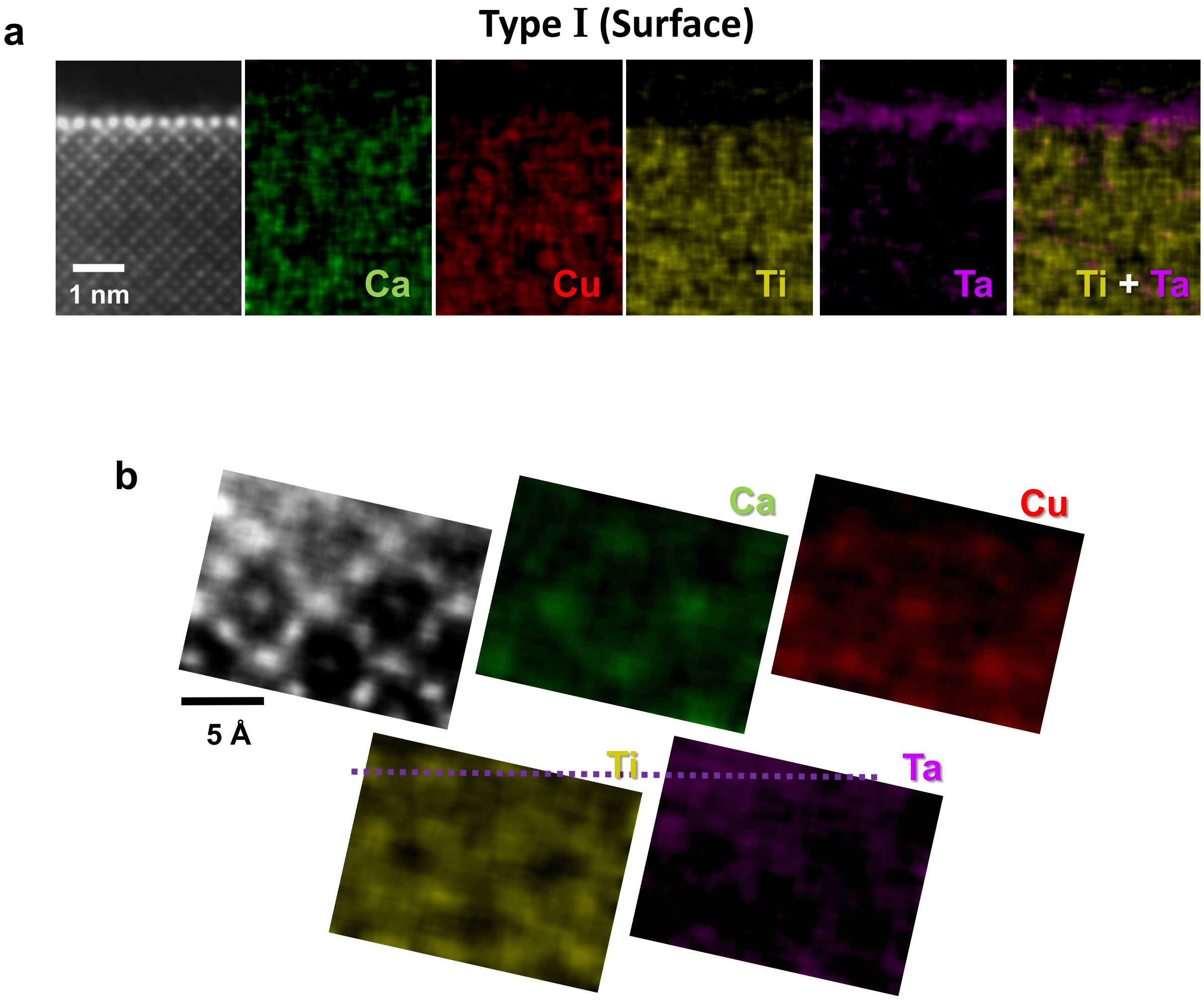

Supplementary Fig. 5 Series of EDS composition maps for Type-I free surface in a Ta-doped sample. **a** The bright-contrast layer shown in the HAADF-STEM image is consistently confirmed to be Ta-rich by the EDS. **b** Atomic-scale EDS maps are provided to directly prove the Ta substitution for the Ti columns in the space-charge segregation layer, as indicated by the dashed purple line. The ordered array of Ca and Cu is also visualized in the atomic-scale maps in the upper row.

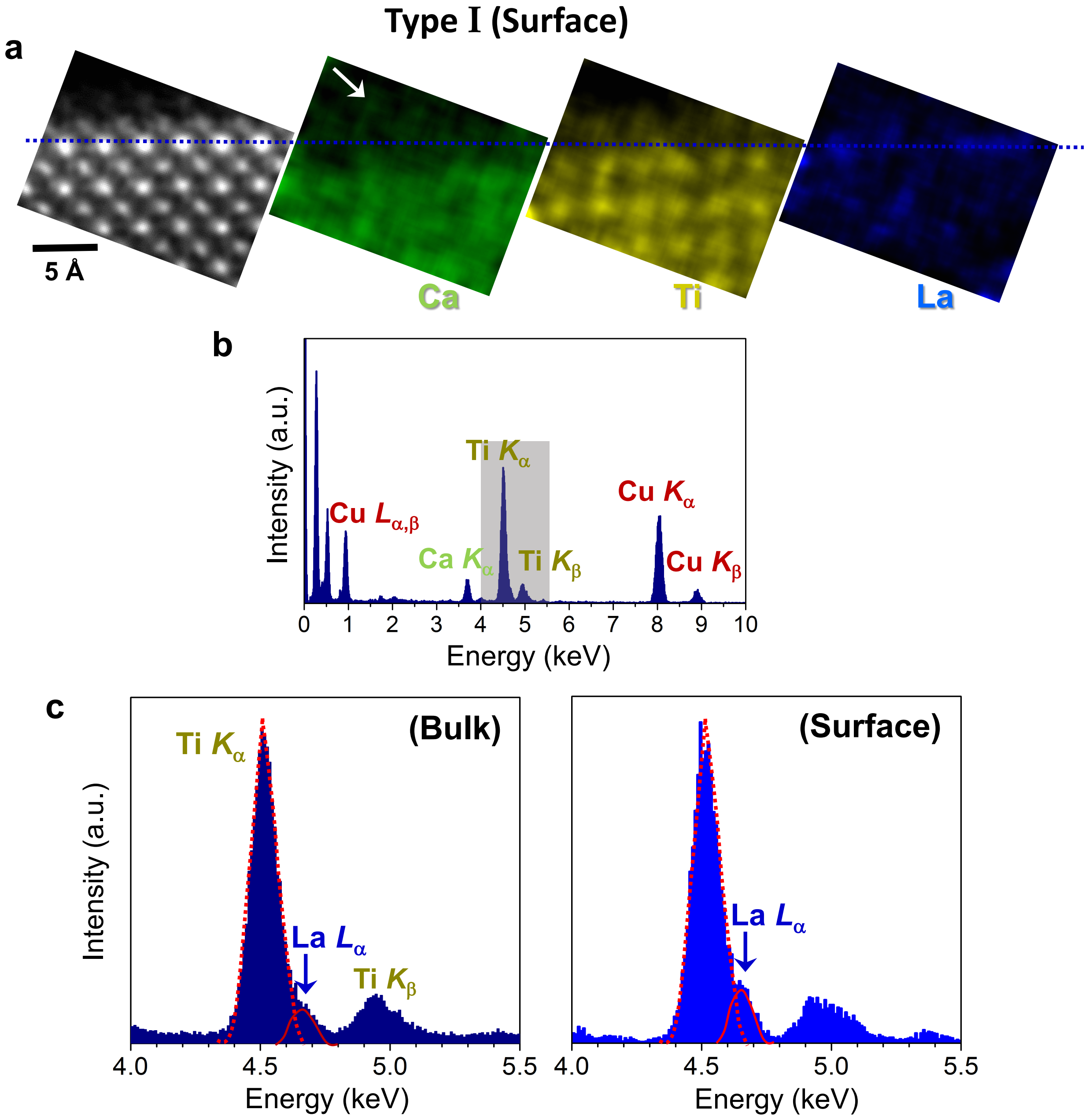

Supplementary Fig. 6 A set of EDS maps for Type-I free surface and comparison of EDS spectra acquired in a La-doped sample. **a** As denoted by a white arrow in the Ca map, the top-most surface is chemically verified to consist of the *A*-site cations including Ca. The blue dashed line denotes the La segregation layer corresponding to *A*-site-cation columns. **b** A typical EDS spectrum acquired in the La-doped sample is provided to show each of the X-ray signal peaks used for atomic-scale mapping. As the La-L_α peak is quite small and close to the Ti-K_α peak in the gray shadow, it is not clearly visible in this overview spectrum. **c** Although the positions of the La-L_α (4.65 keV) and Ti-K_α (4.5 keV) peaks are fairly close to each other, the energy resolution (~0.1 keV) of the EDS detector is sufficient to quantify the intensity of the La-L_α peak. As denoted by red curves on each of the spectra, a higher intensity of the La-L_α peak can be identified in the EDS spectrum from the surface region than from the bulk, verifying the segregation of La.

Type II

(Solid–Solid Heterointerface)

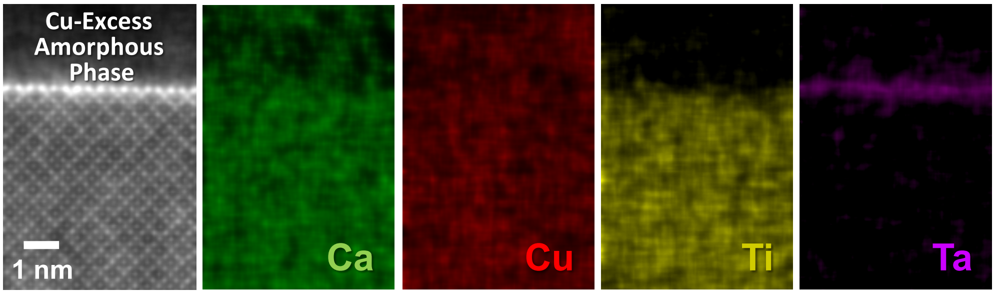

Supplementary Fig. 7 EDS composition maps obtained from a Type-II interface in a Ta-doped sample. This is a solid–solid interface between a grain and a Cu-excess amorphous second phase. Substantial Ta segregation is chemically confirmed by EDS in the Ta map in accord with the bright contrast shown in the HAADF image.

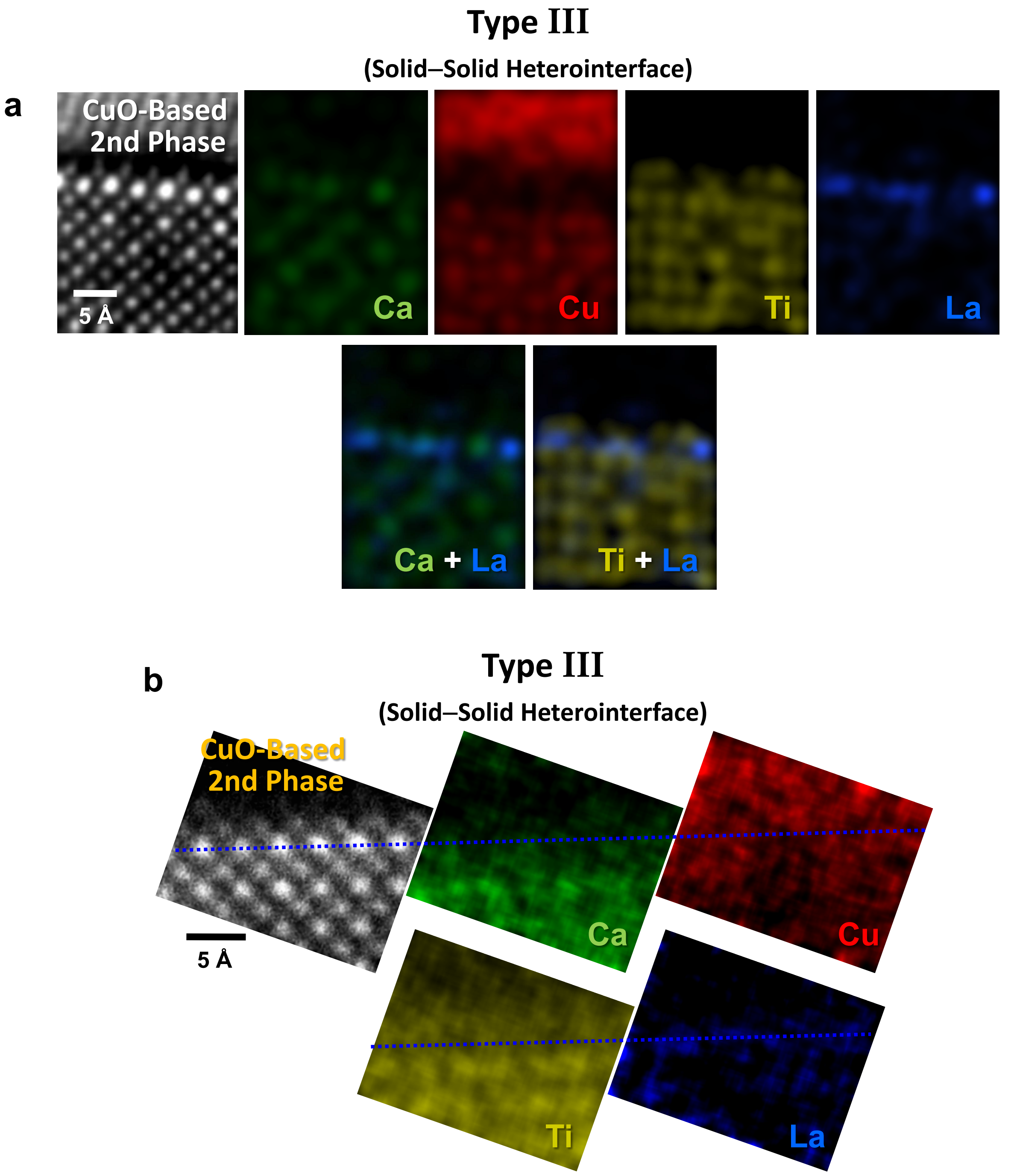

Supplementary Fig. 8 Two sets of atomic-scale EDS maps obtained from Type-III interfaces in a La-doped sample. Type III corresponds to a heterogeneous interface between a grain and a Cu-excess crystalline second phase. **a** Along with the (Ca + La) and (Ti + La) maps in the lower row, La is demonstrated to segregate in the *A*-site layer in the space-charge region. **b** An extra set of atomic-column EDS maps consistently verifies the La segregation with brighter column intensity in the image, as noted by blue dashed lines.

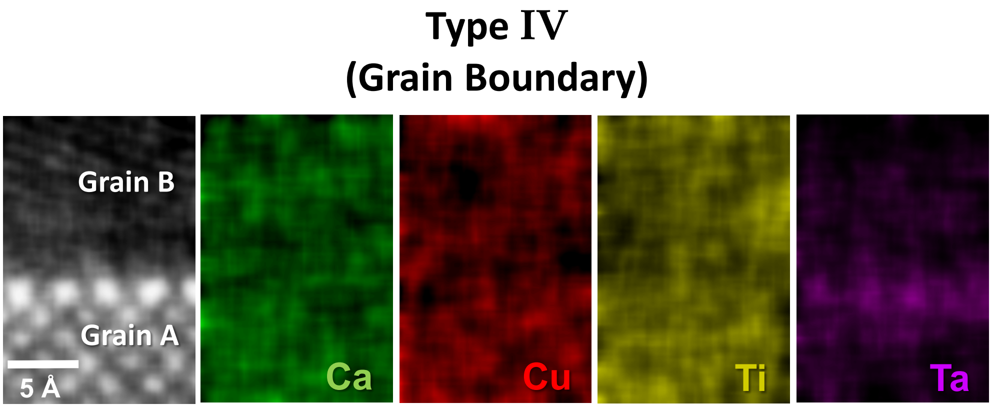

Supplementary Fig. 9 EDS composition maps obtained from a Type-IV grain boundary in a Ta-doped sample. The notable segregation of Ta is clearly visualized at an atomic level between Grains A and B, not in the grain-boundary core but in the neighboring space-charge layer.

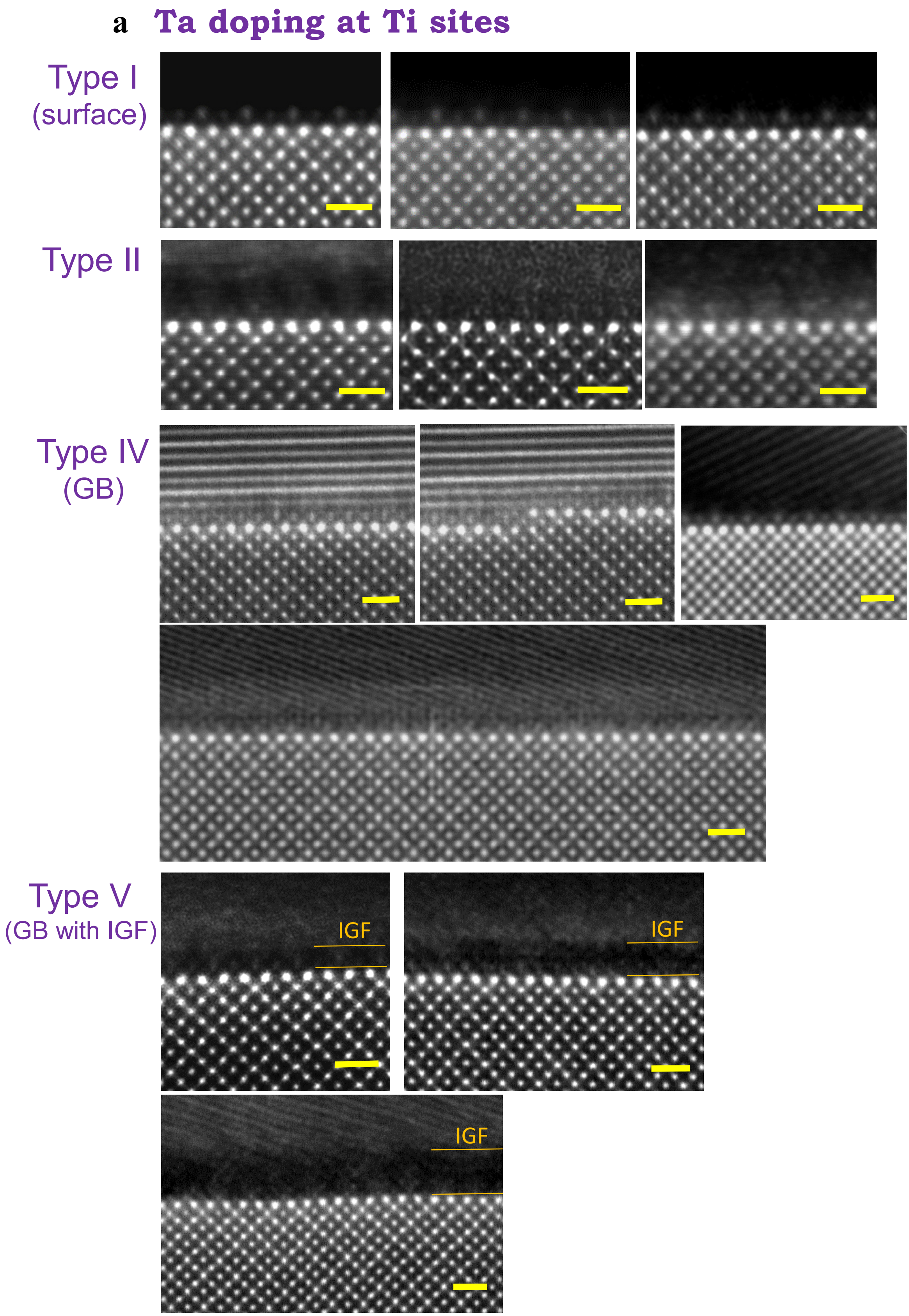

Supplementary Fig. 10 Collection of STEM images. (continued on the next page)

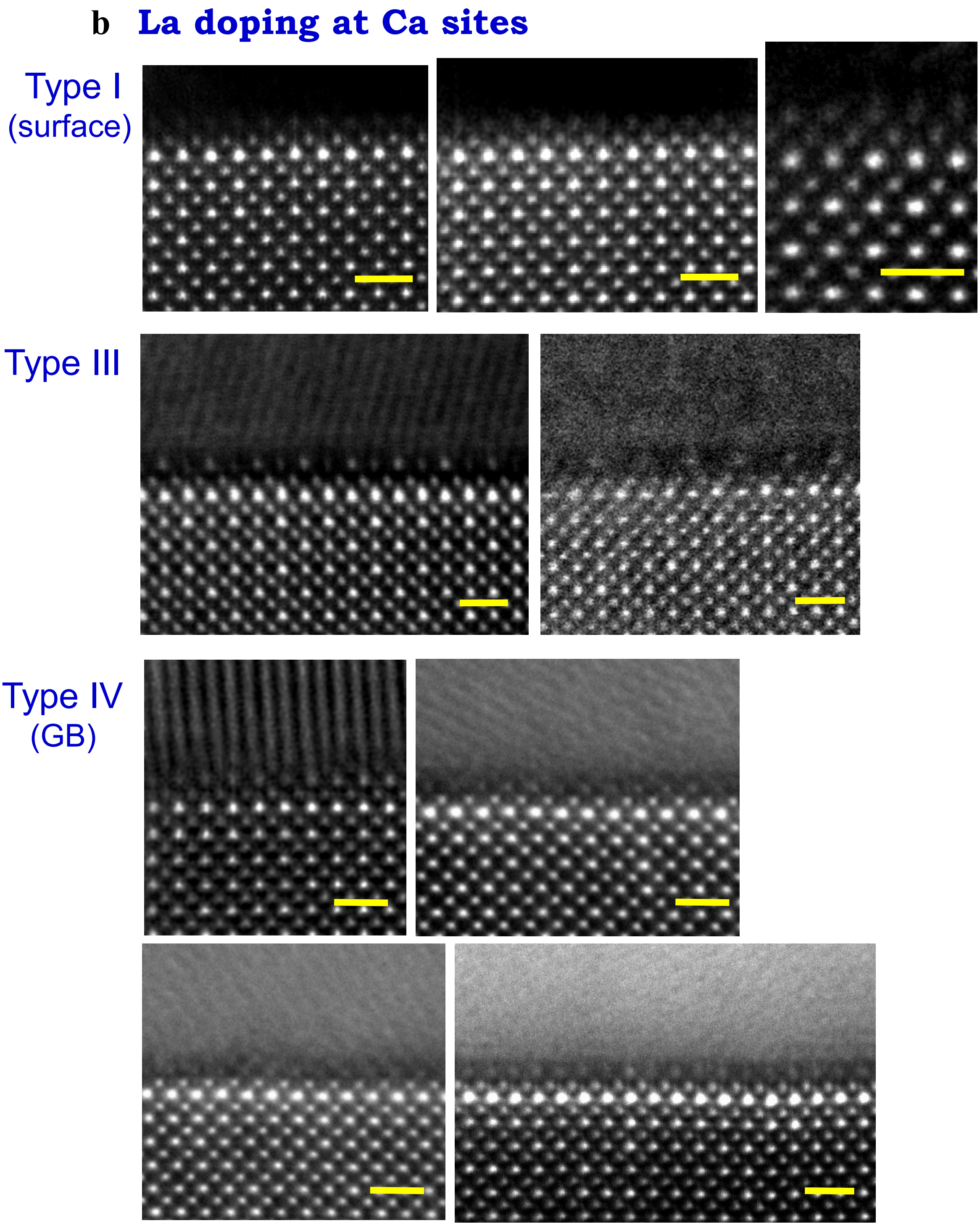

Supplementary Fig. 10 Collection of STEM images. These two sets of image data are a collection of HAADF images acquired for the five different types of interfaces in both the (**a**) Ta- and (**b**) La-doped polycrystalline samples. Scale bars, 7.4 Å (one-unit-cell length).

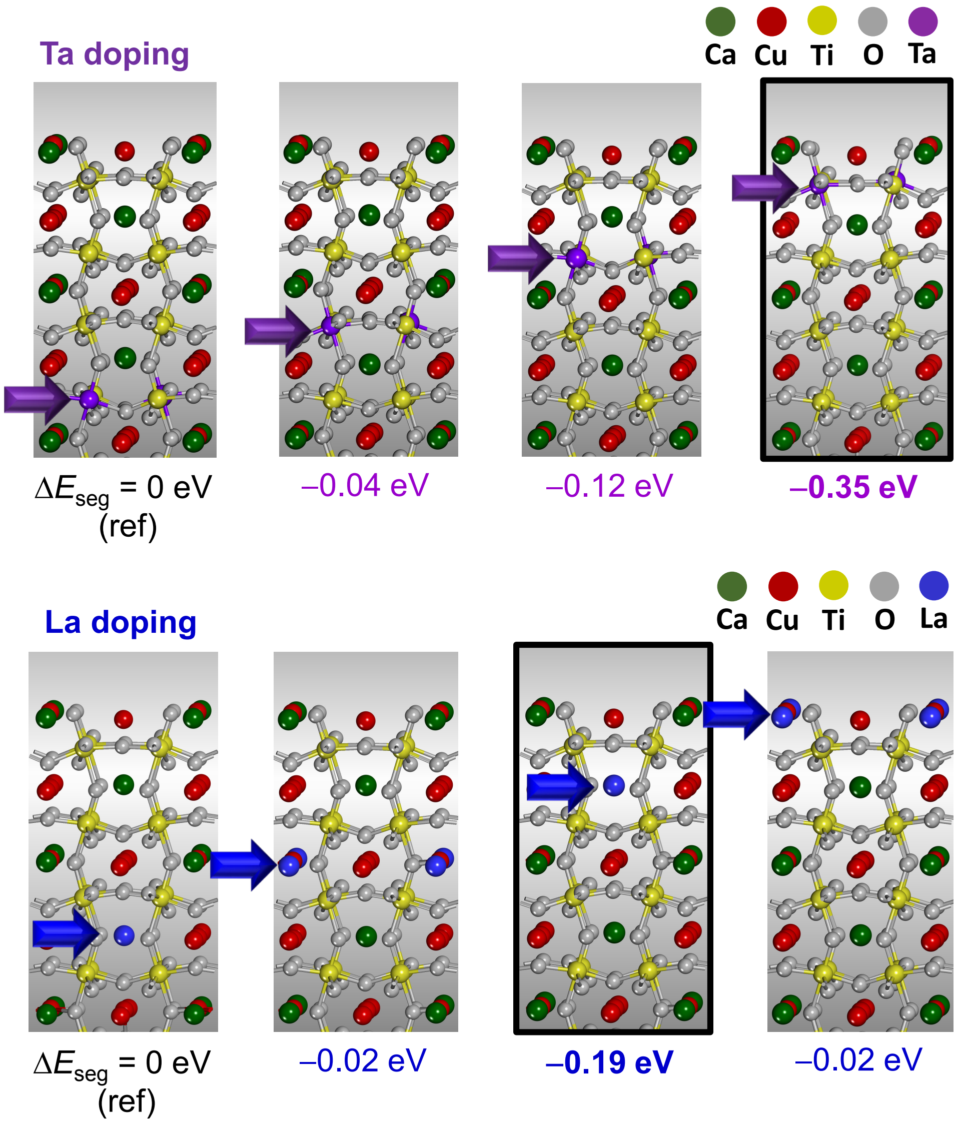

Supplementary Fig. 11 Two sets of DFT calculations. Theses show the relative energy differences with respect to the lattice energy where Ta locates in the fourth subsurface Ti layer (upper panel) and La locates in the fourth subsurface Ca layer (lower panel) below the (100) surface. The energy difference listed here is defined as ΔE_{seg} = E (Ta/La in the nth Ti/Ca layer) – E (Ta in the fourth Ti/Ca layer), where E is the lattice enthalpy of a supercell. As a result, a more negative value of ΔE_{seg} indicates an energetically more favorable configuration of Ta and La. As framed by a black rectangle, most Ta segregates to the first subsurface Ti layer (ΔE_{seg} = –0.35 eV) in good agreement with the STEM analysis in the main text. In addition, ΔE_{seg} shows the lowest value (–0.19 eV), when La locates in the subsurface Ca layer, *not* on the top-most surface, consistently supporting the atomic-scale observation shown in Fig. 3.

Supplementary Note 1

General description

Very helpful details on the ionic space-charge theory along with the electrostatic interactions can be found in several well-documented classic articles by Kliewer and Koehler[1], Poeppel and Blakely[2], and Ikeda and Chiang[3]. Although the mathematical derivation of a series of equations to explain the formation of space charge and the resulting distribution of charged defects near surface is clarified in the literature, we summarize the main equations regarding the space-charge theory for readers.

In this Note, we deal with a *M*O-type oxide composed of divalent metal cations, M^{2+} as a model system. It is also assumed that a pristine *M*O crystal is of one dimension having a free surface at $x = 0$ and also the predominant type of lattice defects is Schottky disorder (a pair of *M* and O vacancies, V_M'' and $V_O^{\bullet\bullet}$ in the Kröger–Vink notation) with no preferential defect binding for simplicity. The following relationship thus is given in *M*O in general.

$$[V_M''](x) = \exp\left\{-\frac{g_{V_M} - 2e\phi(x)}{kT}\right\} \tag{1}$$

$$[V_O^{\bullet\bullet}](x) = \exp\left\{-\frac{g_{V_O} + 2e\phi(x)}{kT}\right\} \tag{2}$$

The bulk value of ϕ_∞ far from the surface can be readily calculated (Eq. 10) by using the charge neutral condition, $[V_M'']_\infty = [V_O^{\bullet\bullet}]_\infty$.

$$g_{V_M} - 2e\phi_\infty = g_{V_O} + 2e\phi_\infty \tag{3}$$

$$e\phi_\infty = \frac{1}{4}\{g_{V_M} - g_{V_O}\} \tag{4}$$

Addition of aliovalent dopants

As can be seen in Eq. 4 above, the electrical potential, ϕ_∞, in the bulk of a pristine *M*O crystal does not have a substantially large value. However, when the crystal is doped with aliovalent cations, a remarkable change of ϕ_∞ is induced, resulting in *significantly varying* $\phi(x)$ in the space-charge region and subsequently strong accumulation/deficiency of dopants and other charged defects near the surface.

For example, if Al^{3+} is extrinsically added as a donor dopant in *M*O and the charge neutrality in the bulk is fully achieved by the formation of cations vacancies, V_M'', their concentration is readily determined by the condition, $2[V_M'']_\infty = [Al_M^{\bullet}]_\infty$. When this equation is combined with Eq. 1, the following relationship is easily deduced for the case of donor doping.

$$e\phi_{\infty} = \frac{g_{V_M}}{2} + kT \ln [Al_{\mathrm{M}}^{\bullet}] - kT \ln 2 \tag{5}$$

Consequently, a very large potential of ϕ_{∞} with a positive sign is constructed in the bulk and thereby rapidly decaying $\phi(x)$ in the space-charge layer is clearly described in the right-hand diagram in Supplementary Fig. 1a. As recognized in Eq. 1, the resultant $[V_M''](x)$ beneath the surface should be thus significantly reduced and the strong accumulation of positively charged $Al_{\mathrm{M}}^{\bullet}$ is accompanied at the same time. The illustration on the left-hand side in Supplementary Fig. 1a depicts such circumstances, demonstrating the space-charge segregation of the donor.

The electrical potential in acceptor doping is also derived in the same manner. For example, when Na^{+} is added as an acceptor dopant in *M*O and the ionic compensation by oxygen vacancies is assumed ($2[V_{\mathrm{O}}^{\bullet\bullet}]_{\infty} = [Na_{\mathrm{M}}']_{\infty}$), the electrical potential in the bulk is given as

$$e\phi_{\infty} = -\frac{g_{V_O}}{2} - kT \ln [Na_{\mathrm{M}}'] + kT \ln 2 \tag{6}$$

In contrast to the donor doping case, a significantly large potential of ϕ_{∞} with a negative sign is verified, resulting in acceptor segregation, as distinctively described in Supplementary Fig. 1b. Thus, the concentrations of both types of dopants can be expressed in general as the exponentially varying simple Boltzmann distribution (Eq. 7), as noted in Ref. 3:

$$\frac{n_i(x)}{n_{i,\infty}} = \exp\left\{\frac{-z_i e[\phi(x) - \phi_{\infty}]}{kT}\right\} \tag{7}$$

where n_i and z_i are the concentration and the effective charge of a dopant, respectively.

Supplementary References

1. Kliewer, K. L. & Koehler, J. S. Space charge in ionic crystals. I. General approach with application to NaCl. *Phys. Rev.* **140**, A1226–A1240 (1965).
2. Poeppel, R. B. & Blakely, J. M. Origin of equilibrium space charge potentials in ionic crystals. *Surf. Sci.* **15**, 507–523 (1969).
3. Ikeda, J. A. S. & Chiang, Y.-M. Space charge segregation at grain boundaries in titanium dioxide: I, Relationship between lattice defects chemistry and space charge potential. *J. Am. Ceram. Soc.* **76**, 2437–2446 (1993).
